# Developmental transcriptome of resting cell formation in *Mycobacterium smegmatis*

**DOI:** 10.1186/s12864-016-3190-4

**Published:** 2016-10-26

**Authors:** Mu-Lu Wu, Martin Gengenbacher, Jade C. S. Chung, Swaine Lin Chen, Hans-Joachim Mollenkopf, Stefan H. E. Kaufmann, Thomas Dick

**Affiliations:** 1 Antibacterial Drug Discovery Laboratory, Department of Microbiology and Immunology, Yong Loo Lin School of Medicine, National University of Singapore, Singapore, Singapore; 2 Tuberculosis Research Laboratory, Department of Microbiology and Immunology, Yong Loo Lin School of Medicine, National University of Singapore, Singapore, Singapore; 3 Department of Medicine, Division of Infectious Diseases, Yong Loo Lin School of Medicine, National University of Singapore, National University Health System, Singapore, Singapore; 4 Infectious Diseases Group, Genome Institute of Singapore, Singapore, Singapore; 5 Core Facility Microarray / Genomics, Max Planck Institute for Infection Biology, Berlin, Germany; 6 Department of Immunology, Max Planck Institute for Infection Biology, Berlin, Germany

**Keywords:** SMRC, LARC, Actinobacteria, Sporulation, Evolution

## Abstract

**Background:**

Mycobacteria, along with exospore forming *Streptomyces*, belong to the phylum actinobacteria. Mycobacteria are generally believed to be non-differentiating. Recently however, we showed that the mycobacterial model organism *M. smegmatis* is capable of forming different types of morphologically distinct resting cells. When subjected to starvation conditions, cells of *M. smegmatis *exit from the canonical cell division cycle, segregate and compact their chromosomes, and become septated and multi-nucleoided. Under zero nutrient conditions the differentiation process terminates at this stage with the formation of Large Resting Cells (LARCs). In the presence of traces of carbon sources this multi-nucleoided cell stage completes cell division and separates into Small Resting Cells (SMRCs). Here, we carried out RNA-seq profiling of SMRC and LARC development to characterize the transcriptional program underlying these starvation-induced differentiation processes.

**Results:**

Changes among the top modulated genes demonstrated that SMRCs and LARCs undergo similar transcriptional changes. The formation of multi-nucleoided cells (i.e. LARCs and the LARC-like intermediates observed during SMRC formation) was accompanied by upregulation of septum formation functions FtsZ, FtsW, and PbpB, as well as the DNA translocase FtsK. The observed compaction of chromosomes was accompanied by an increase of the transcript level of the DNA binding protein Hlp, an orthologue of the *Streptomyces* spore-specific chromosome condensation protein HupS. Both SMRC and LARC development were accompanied by similar temporal expression patterns of candidate regulators, including the transcription factors WhiB2, WhiB3, and WhiB4, which are orthologues of the *Streptomyces* sporulation regulators WhiB, WhiD and WblA, respectively.

**Conclusions:**

Transcriptional analyses of the development of mycobacterial resting cell types suggest that these bacteria harbor a novel differentiation program and identify a series of potential regulators. This provides the basis for the genetic dissection of this actinobacterial differentiation process.

**Electronic supplementary material:**

The online version of this article (doi:10.1186/s12864-016-3190-4) contains supplementary material, which is available to authorized users.

## Background

Mycobacteria distribute broadly across environments, including fresh water, municipal water systems and soil [1], where they encounter nutrient limitation frequently. This group of actinobacteria is known for its extraordinary capacity to survive for years under starvation conditions [2]. A laboratory starvation culture model based on saline with zero nutrients was established by Loebel and his colleagues in the 1930s [3, 4]. Upon starvation in this model, the bacilli terminate growth and enter a non-replicating state without any apparent morphological changes, but with reduced metabolism. Furthermore, the non-replicating bacteria display dramatic phenotypic drug resistance [5–7]. Recently we showed for *Mycobacterium smegmatis* that starvation in saline containing traces of a carbon source, as opposed to shock starvation in zero nutrient saline, triggers the development of mono-nucleoided Small Resting Cells, SMRCs. Formation of SMRCs occurred via a septated multi-nucleoided intermediate. Shock starvation in nutrient-free saline resulted as expected in apparently unaltered log-phase-sized resting cells we termed Large Resting Cells, LARCs. Surprisingly, fluorescence microscopic analyses revealed that LARCs remodeled their interior to form septated multi-nucleoided cells similar to the cell intermediate observed during SMRC development. In contrast to growing bacilli, both SMRCs and LARCs had condensed nucleoids, reduced metabolism and increased stress and antibiotic tolerance. In the first weeks of starvation, SMRCs and LARCs showed similar survival. SMRCs however, displayed an increased long-term survival when starvation was extended to 6 months, which correlated with their ability to retain intracellular lipid bodies as energy storage. Based on the morphological similarity between LARCs and the septated multi-nucleoided, LARC-like cell intermediates observed during SMRC development, we hypothesized that mycobacteria (i) undergo a previously unknown differentiation into SMRCs and LARCs; (ii) SMRCs develop through a LARC-like stage; and (iii) formation of SMRCs and LARCs should demonstrate distinct transcriptional profiles compared with non-starved mycobacteria [8]. Here, we used RNA-seq to profile the transcriptomes of *M. smegmatis* during SMRC and LARC development.

## Results

### Experimental setup for RNA-seq analyses

**Fig. 1:**
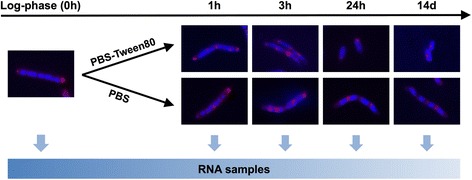
Illustration of samples collected for RNA-sequencing. Development of small and large resting cells in PBS-Tween80 and PBS was demonstrated using DAPI (*blue*, to visualize DNA) and FM4-64 (*red*, to visualize membranes) stained culture samples. As reported previously [8], 1 h and 3 h starved cultures under both shock and gentle starvation conditions formed intracellular septa; at 24 h after starvation, the formation of SMRCs and LARCs was largely completed. Samples for RNA-sequencing were collected accordingly at these critical cellular differentiation time points to determine the corresponding developmental transcriptome profiles. Log-phase samples were collected as baseline control and 14-day-old starved cultures were sampled to reflect long-term transcriptional changes. Note: although it has been suggested before that membrane and septal peptidoglycan co-localized at septal positions in dividing mycobacterial cells [41], the formation of peptidoglycan at septal positions in the starved cells remains to be determined

Exponentially growing *M. smegmatis* in rich 7H9 broth (0 h sample, baseline) was transferred to either phosphate buffered saline (PBS) only, or PBS containing traces of Tween80, an oleic acid ester that is used by mycobacteria as carbon source [9]. RNA samples were collected for transcriptional profiling at the following time points to capture the transcriptomes of the critical developmental stages as illustrated in Fig. 1 [8]: after 1 h and 3 h of starvation to capture immediate responses to nutrient limitation; and after 24 h when LARC formation in PBS cultures and SMRC formation in PBS-Tween80 cultures are largely completed. Fourteen-day-old starved cultures were sampled to determine long-term gene expression profiles. Genes were considered statistically significant differentially expressed when their transcript level was at least 2-fold up- or down-regulated compared to the 0 h baseline (*P*-value < 0.001). A list of all significantly differentially expressed genes for each time point under PBS- or PBS-Tween80-starvation conditions can be found in Additional file 1: Table S1.

### Global transcriptional responses to shock and gentle starvation

**Fig. 2:**
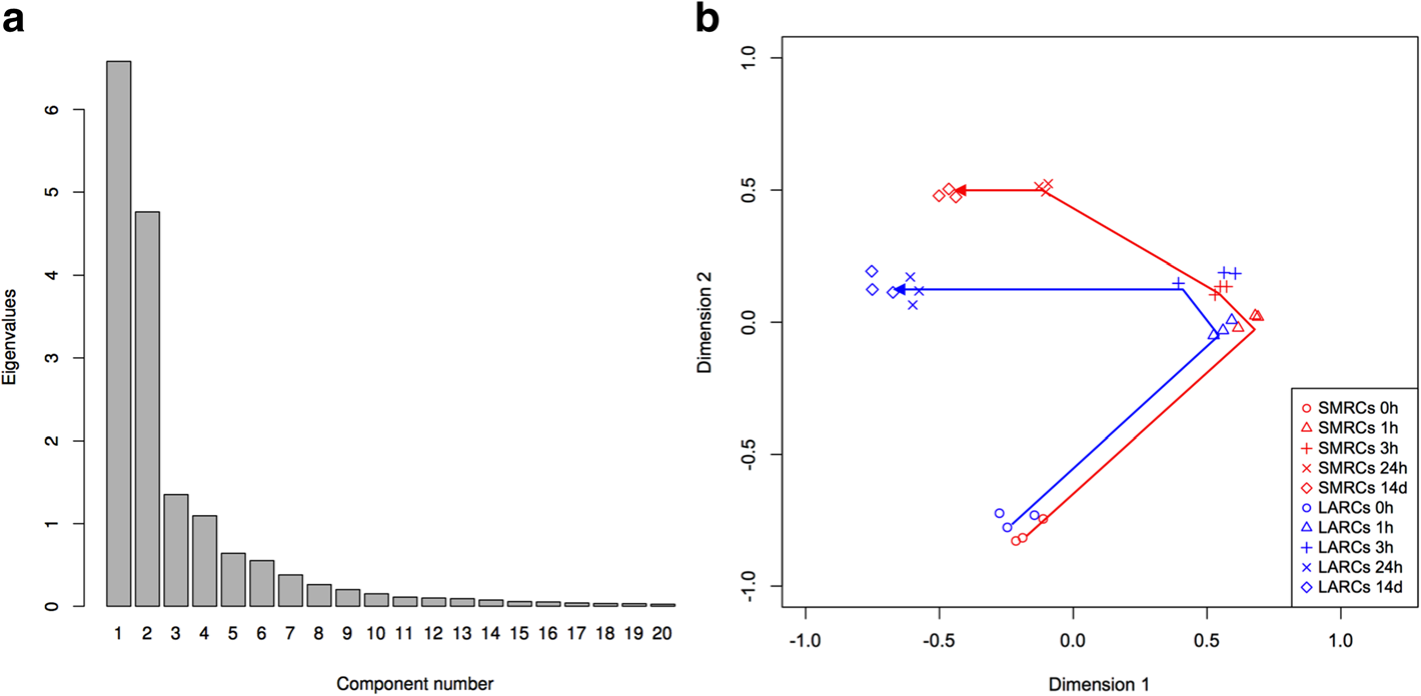
Overview of transcriptional changes. **a** Scree plot of eigenvalues for the dimensions extracted from a multidimensional scaling (MDS) analysis of the 500 genes with the highest variations in expression during LARC and SMRC development. Dimension 1 accounted for 45 % of the total variance, while dimension 2 accounted for 32 % of the total variance. **b** Projection of samples onto the first two MDS dimensions. Each sample, representing a single biological replicate of a single culture under one growth condition and one time point, is represented by a single symbol. Samples grown with trace carbon (leading to SMRCs) are colored red, while those with no carbon source (leading to LARCs) are depicted in blue. Different symbols indicate different time points as indicated in the legend at the bottom right. Points with same color and symbol represent the three biological replicates for the RNA-seq analyses. Arrows indicate the development of SMRCs and LARCs over time (0, 1, 3, 24 h and 14 d)

Overall, a relatively large fraction (~1/3) of genes showed significant changes in abundance in at least one time point during the 2-week study period (see Additional files 2: Tables S2 and Additional files 3: Table S3, Additional files 4: Figure S1 and Additional files 5: Figure S2). To examine relationships between overall transcriptional patterns among the individual time points, we used multidimensional scaling (MDS) on the 500 genes with the highest variation in expression (at any time point or growth condition in our dataset). The scree plot of variance (Fig. 2a) demonstrates that the first two dimensions of the MDS capture 77 % of the total variance in expression among these 500 genes; we therefore focused the rest of our analysis on these first two dimensions, which can be conveniently displayed in a scatter plot (Fig. 2b). All the log-phase samples were “close” to each other in this scatter plot, indicating that they were generally similar in their expression of these 500 genes. The 1 h and 3 h starved cultures (regardless of carbon source availability), in which the bacilli under both starvation conditions terminate growth and start septum formation, chromosome segregation and condensation to form septated multi-nucleoided cells (Fig. 1), are transcriptionally similar. 24 h starved cultures, when bacilli under both starvation conditions have mostly completed their microscopically observable differentiation into LARCs and SMRCs, show distinct transcriptional profiles. Consistent with the cell biological observations suggesting that differentiation is largely completed within 24 h after the onset of starvation, expression profiles between cells starved for 24 h and 14 d are reasonably similar in this visualization. There is a small shift in the transcriptional profiles from 24 h to 14 d for SMRCs, which may indicate additional developmental changes after formation of the mono-nucleoided cells which are not microscopically observable. In contrast, the 24 h and 14 d LARC samples are closely clustered; further examination of similar plots out to MDS dimension 5 (accounting for a total of >95 % of total variance) shows that these samples remained clustered as closely as the biological replicates of the 0 h samples (data not shown).

Taken together, the overall transcriptional profiles therefore suggest a similar genetic program in the early phase of LARC and SMRC formation, consistent with a common response to starvation and the formation of septated multi-nucleoided cells under both starvation conditions. After 24 h, when the microscopically observable differentiation into LARC and SMRC is largely completed, the respective transcriptional patterns are distinct and do not change in an appreciable way over the next 2 weeks.

### Comparative transcriptional responses of selected genes

**Fig. 3:**
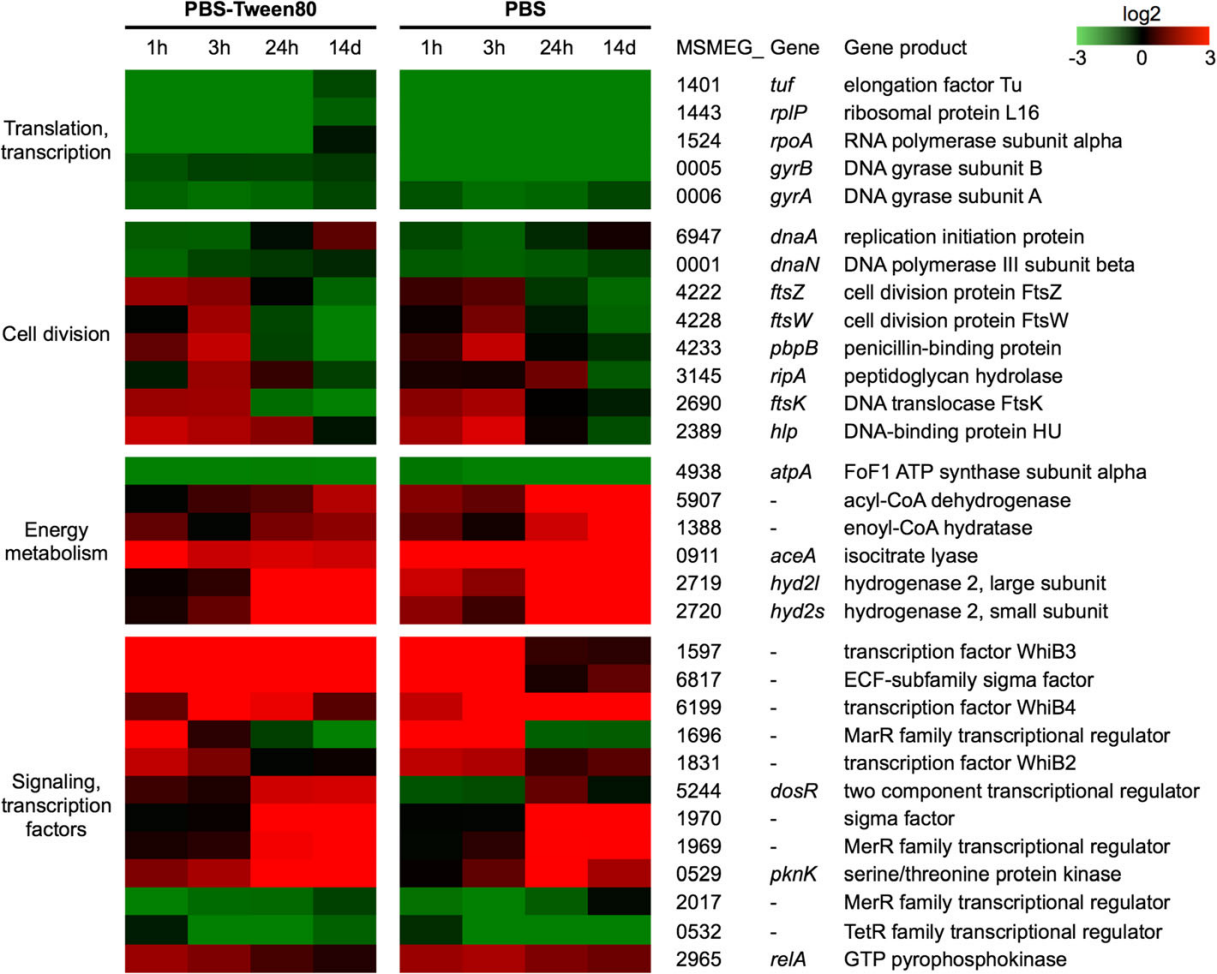
Transcript changes over time of representative genes in PBS-Tween80- and PBS-starved *M. smegmatis* cultures. A heat map denoting upregulated genes in red and downregulated genes in green is shown. Quantifications for the genes shown (and transcript changes of all statistically significant differentially expressed genes) are in Additional file 1: Table S1

Figure 3 depicts changes of transcript levels for several individual genes over time to illustrate the largely similar kinetics in shock and gently starved cultures, allowing us to generate hypotheses regarding the regulatory program within these cells. Translation (elongation factor Tu, ribosomal protein L16) and transcription (RNA polymerase subunit alpha, DNA gyrase) functions, as well as DNA replication functions (replication initiation protein DnaA, DNA polymerase subunit beta DnaN) were immediately downregulated, consistent with the observed starvation-induced termination of growth.

Intriguingly, an upregulation of *ftsZ*, *ftsW* and *pbpB* was observed during the first 3 h in the starved non-growing cultures compared to exponentially growing cells. The corresponding proteins encoded by these three genes—Z ring protein FtsZ, cell division protein FtsW and penicillin-binding protein PpbB—have been shown to interact and form a ternary septation complex in mycobacteria which is involved in septum synthesis, especially in septal peptidoglycan biogenesis [10]. The upregulation of these septum formation functions is consistent with the observed onset of intracellular septum formation in LARC and SMRC cultures during the early hours upon starvation (Fig. 1). Concurrently the transcript level of FtsK, a DNA translocase involved in translocating any residual DNA [11], was also increased. Additionally, expression of RipA, a peptidoglycan hydrolase involved in the physical separation of daughter cells [12], was significantly upregulated during SMRC formation at 3 h.

Noteworthy is an increase in the transcript level of the histone-like DNA binding protein Hlp, associated with compaction of DNA [13, 14], mirroring the nucleoid condensation observed in LARCs and SMRCs shown in Fig. 1 [8]. Interestingly, Hlp upregulation was also observed previously when *M. smegmatis* shifted down to a non-replicating state induced by oxygen deprivation in nutrient rich medium [15].

Genes involved in energy metabolism were largely downregulated as illustrated by the reduction in mRNA level of ATP synthase subunits, e.g. AtpA, consistent with the observed reduced ATP level and oxygen consumption rate observed in both types of starved cultures [8]. Consistent with the observed higher utilization of fat storage bodies in LARCs when compared to SMRCs [8], upregulation of transcripts encoding β-oxidation enzymes (acyl-CoA dehydrogenase and enolyl-CoA hydratase) was more pronounced in LARCs. In line with the shift to catabolism of fatty acids, expression of isocitrate lyase AceA involved in the glyoxylate shunt [5] was increased under both starvation conditions. Interestingly, the genes encoding the subunits of the hydrogenase Hyd2, were strongly induced in both starved cultures from 24 h onwards (Fig. 3). These gene products were recently shown to be upregulated in growing carbon-limited *M. smegmatis* cultures and to oxidize atmospheric H_2_, thus providing electrons to the electron transport chain for ATP generation [16].

Differentiation into LARCs and SMRCs presumably requires changes in signaling and transcription factors. Based on the overlapping cellular differentiation pathways suggested by the microscopic analyses (Fig. 1) and the similarities in gene expression changes over time (Fig. 2b), we expected largely overlapping patterns in the expression of regulatory genes. Figure 3 (*bottom*), depicting the changes in transcript levels of selected strongly modulated regulatory genes, demonstrates that this is indeed the case—with some noteworthy variation between the two developmental pathways. The expression of the transcription factor WhiB3 and an ECF-subfamily sigma factor was immediately and profoundly induced (20- to 160-fold, see Additional file 1: Table S1). Whereas SMRC/PBS-Tween80 cultures maintained a high transcript level throughout (i.e. until 14 d), the two genes were downregulated from 24 h onwards in LARC/PBS cultures. In contrast, another transcription factor, WhiB4, was strongly induced throughout in LARC/PBS cultures, but more transiently upregulated in SMRC/PBS-Tween80 cultures. Other early response transcription factors were a member of the MarR family and WhiB2. These genes were only transiently upregulated during the first 1–3 h in both cultures. Late transcriptional regulators that were only strongly upregulated from 24 h onwards, i.e. after cellular differentiation was largely completed, were also identified. Examples are the dormancy survival response regulator DosR, shown previously to be involved in the survival of oxygen-depletion induced non-replicating cultures [17], another sigma factor and a MerR family regulator (Fig. 3). Another late starvation function appears to be the serine protein kinase gene *pknK*, suggesting a possible role of protein phosphorylation in the adaptation to starvation-induced non-replicating survival. Also shown in Fig. 3, other transcription factors, such as another MerR and a TetR family regulator were profoundly (20- to 50-fold) downregulated upon starvation. Finally, as expected, transcript abundance of the stringent response factor RelA was upregulated under both starvation conditions. Previously it was shown that the stringent response is required for starvation survival in vitro and persistence of *Mycobacterium tuberculosis* in animal models [18–21].

Taken together, the largely similar transcript level changes in PBS- and PBS-Tween80-starved cultures are consistent with the observed similarities in cellular differentiation events and physiological changes of *M. smegmatis* under both starvation conditions [8]. The dynamic transcript level changes of regulator genes suggest the presence of a starvation-induced genetic differentiation program.

## Discussion

**Fig. 4:**
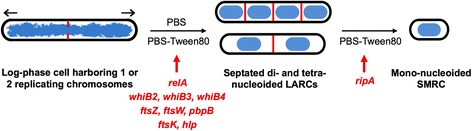
Nutrient-starvation induced differentiation in *M. smegmatis*. Model depicting starvation induced differentiation of log-phase cells into LARCs and SMRCs. Under zero-nutrient starvation (PBS), the development terminates at the septated, multi-nucleoided LARC stage. In the presence of traces of a carbon source (PBS-Tween80), LARCs undergo cell division and separate into SMRCs. Representative genes upregulated early upon starvation are indicated (as shown in Fig. 3). Blue: DNA, red: septa, black: cell envelope. Arrows indicate polar growth of log-phase cells

Upon starvation, *M. smegmatis* is capable of forming at least two morphologically distinct types of resting cells. Starvation in PBS containing traces of a carbon source triggers the development of mono-nucleoided Small Resting Cells, SMRCs. Starvation in PBS with zero carbon source results in the formation of Large Resting Cells, LARCs, which show internal remodeling to septated multi-nucleoided cells similar to the intermediates observed during SMRC development. Based on these similarities we had previously proposed a model in which nutrient starvation with or without traces of a carbon source triggers a differentiation program that results in the formation of septated multi-nucleoided cells [8]. Under zero nutrient conditions the differentiation process terminates at this stage with the formation of LARCs. In the presence of traces of carbon sources this septated multi-nucleoided cell stage completes cell division and separates into SMRCs (Fig. 4). We have now shown by transcriptional profiling that morphological changes during SMRC and LARC development are generally mirrored by large-scale changes in gene transcription. In particular, early stages of starvation with or without trace carbon result in similar morphological and genetic changes in *M. smegmatis*, while the later stages (24 h and beyond) that are morphologically distinct are also transcriptionally distinct. The formation of LARCs and the septated multi-nucleoided LARC-like intermediates observed during SMRC formation is accompanied by the upregulation of the septum formation functions FtsZ, FtsW, and PbpB, as well as the DNA translocase FtsK. Compaction of chromosomes is accompanied by an increase of the transcript level of the histone binding protein Hlp. Both SMRC and LARC development is accompanied by similar temporal expression patterns of candidate transcriptional regulators. Taken together, the correlated morphological and transcriptional changes strongly suggest that mycobacteria harbor a previously unrecognized differentiation program activated by starvation. We have further identified several potential regulators that may provide further insight into resting cell development.

Transcriptome profiles of nutrient-limited *growing M. smegmatis* in continuous cultures have been studied previously [22, 23]. As reported in those studies [22], we also observed marked adaptations in energy metabolism, including upregulation of glyoxylate shunt pathway (e.g. isocitrate lyase AceA) and activation of alternative hydrogenases (e.g. hydrogenase Hyd2) for energy generation. However, consistent with the fact that the carbon-limited continuous cultures were growing, DNA replication functions (e.g. replication initiation protein DnaA, DNA polymerase subunit DnaN) which were immediately downregulated in our non-growing cultures, showed no significant transcriptional changes in the previously reported nutrient-limited growing cells [22]. Moreover, key genes and potential regulators such as *ftsZ*, *ftsW*, *pbpB*, *ftsK* and *whiB3* that were upregulated dramatically under our starvation conditions, showed no differential expression in continuous, carbon-limited growing cultures. Overall, our data are generally consistent with previously reported transcriptome data for growing nutrient-limited mycobacterial cultures. Additionally, using a batch culture starvation system generating non-growing bacteria, we identified a novel transcriptional differentiation program that governs the recently uncovered starvation-induced morphological differentiation processes, i.e. SMRC and LARC formation.

It is interesting to note that orthologues of some of the genes upregulated during the formation of mycobacterial resting cells are involved in the formation of exospores (another distinct type of resting cell), in *Streptomyces. Streptomyces*, like *Mycobacterium*, belongs to the phylum actinobacteria. The two bacterial groups separated about 1.3 billion years ago [24, 25]. In response to nutrient depletion, *Streptomyces* initiates growth of aerial hyphae. These multi genomic structures septate to form chains of mono-nucleoided pre-spore compartments, and then break up into individual small resting cells called exospores [26]. Two categories of genes, *bld* and *whi*, have been shown to be the key developmental regulators for the exosporulation process [27]. Interaction among Bld proteins, e.g. BldN, BldD, BldH, has been shown to be important in repressing vegetative growth and initiating the formation of aerial mycelium [27–30]; while five central *whi* genes—*whiA*, *whiB*, *whiG*, *whiH* and *whiI*—are critical for early stages of the conversion from aerial hyphae to spores [27, 31, 32]. Besides these central Whi family regulators, additional WhiB-like transcription factors including WhiD and WblA, are required for morphological differentiation in *Streptomyces* for the placement of septa and spore maturation [33, 34]. The majority of the sporulation-specific *Streptomyces* transcriptional regulators have no orthologues in mycobacterial genomes. However interestingly, mycobacterial orthologues of sporulation specific *Streptomyces* WhiB, WhiD and WblA—WhiB2, WhiB3, and WhiB4 [24]—were strongly upregulated during the development of SMRCs/LARCs. Apparent molecular similarities between mycobacterial resting cell formation and *Streptomyces* exospore formation also extend to some of the key functional sporulation genes. The mycobacterial orthologue of the *Streptomyces* DNA translocase FtsK, which mobilizes chromosomal DNA through the closing septum in sporulating aerial hyphae [35], was upregulated during mycobacterial resting cell formation. The mycobacterial orthologue of the *Streptomyces* sporulation-specific DNA-packaging protein HupS [36], Hlp, was also found to be upregulated during LARC/SMRC formation. It is interesting to note that an additional domain in HupS/Hlp, which makes the DNA binding protein unusual compared the canonical HU proteins in bacteria, can only be found in mycelial actinobacteria and *Mycobacterium* spp. [24]. Although SMRC formation in mycobacteria and exospore formation in *Streptomyces* are obviously different processes, the similarities we have observed in the cell biology (formation of septated multi-nucleoided intermediates followed by the separation of mono-nucleoided resting cells) and gene transcription during both differentiation processes may hint at an ancient conserved regulatory program that has diverged to produce the distinct resting cell types, SMRCs/LARCs and exospores in modern actinobacteria.

## Conclusions

Overall, our comparative developmental transcriptome studies suggest that *M. smegmatis* harbors a novel transcriptional differentiation program that governs a starvation induced morphological differentiation process. The identification of a set of differentially expressed regulatory genes provides the basis for a reverse genetic approach for the functional dissection of the regulatory program underlying the development of nutrient starvation induced mycobacterial resting cells.

## Methods

### Bacterial strain and culture conditions

*Mycobacterium smegmatis* mc^2^155 (ATCC 700084) obtained from the American Type Culture Collection (Manassas, Virginia, USA) was cultivated in Middlebrook 7H9 broth supplemented with 0.5 % bovine albumin, 0.2 % glucose, 0.085 % NaCl, 0.5 % glycerol, 0.0003 % catalase and 0.05 % Tween80 at 37 °C with agitation. In nutrient starvation experiments, log-phase cultures with an optical density at 600 nm (OD_600_) of 0.5 were pelleted by centrifugation at 25 °C/3200 rpm for 10 min. The harvested cells were then washed three times with PBS-0.025 % Tween80 or PBS-0.025 % Tyloxapol (PBS, Gibco 14080-055) and diluted in the corresponding starvation media to a final OD_600_ of 0.10–0.15. 100 ml of this suspension was then transferred into a 1 L roller bottle (Corning, COR430195) and starved for 14 days (rolling at 2 rpm, 37 °C).

### FM4-64, DAPI staining and fluorescence microscopy

For membrane and DNA staining, samples collected at different time points were first fixed in 2 % paraformaldehyde in PBS for 30 min, washed with PBS and then stained as previously described [8] with membrane dye FM4-64 (Molecular Probes, T3166) at a 1 μg/ml for 1 h, followed by staining with DAPI (Molecular Probes, D1306) at 10 μg/ml for 10 min. For visualization, dual stained samples were mounted to slides and observed under epifluorescence microscope Olympus BX60 using U-M61002 and U-MWIG filters for DAPI and FM4-64, respectively.

### RNA isolation and preparation

RNA samples from 3 biological replicates were collected for transcriptional profiling at each time point. Briefly, 10 ml of exponentially growing cultures at OD_600_ of 0.5 (t = 0) or 50 ml of starved cultures at 1 h, 3 h, 24 h and 14 days were harvested by centrifugation (25 °C, 3200 rpm, 10 min). The pellet was resuspended in 1 ml of Trizol (Invitrogen). Bacterial cultures in Trizol were then disrupted by bead beating (FastPrep Instrument and BLUE Kit tubes (BIO 101); two cycles of 30 s at maximum speed with cooling on ice between cycles). The samples were centrifuged for 1 min at 4 °C/13,000 rpm and the supernatant was transferred to a new tube containing 200 μl chloroform, mixed and incubated at RT for 5 min. After centrifugation at 4 °C/13,000 rpm for 10 min, RNA was extracted from the aqueous phase with isopropanol according the supplier’s manual followed by a treatment with DNase I, Amplification Grade (Invitrogen) treatment with subsequent ammonium acetate / isopropanol precipitation. Quality and quantity of total RNA were assessed using an Agilent 2100 Bioanalyzer (Agilent Technologies) and a NanoDrop 1000 spectrophotometer (Kisker). The Gram-Positive Bacteria Ribo-Zero (Epicentre) rRNA Magnetic Removal Kit was used to remove bacterial rRNA from total RNA preparations.

### RNA sequencing and transcriptome analysis

RNA-seq libraries were prepared according to the TruSeq RNA Sample Preparation v2 Guide (Illumina) without fragmentation and without size selection. Using an Agilent Bioanalyzer high sensitivity DNA kit, we confirmed an average final library size approximately 350 bp rendering fragmentation obsolete. Therefore the library insert fragmentation time at 94 °C was set to 0 min. The quality of the cDNA libraries was assessed using the DNA-1000 kit (Agilent Technologies) on a 2100 Bioanalyzer and quantified with the Qubit 2.0 Fluorometer (Life Technologies). Two libraries of 15 different samples each were indexed, pooled, loaded and clustered onto individual lanes of a Rapid Run flowcell with a cBot instrument (Illumina). In total 30 different samples were analyzed on a Hi-Seq 1500 sequencer (Illumina) using a TruSeq Rapid SBS Kit and 2 × 51 cycles including 7 cycles indexing to obtain 50-bp paired-end reads.

RNA sequencing reads were mapped to the *M. smegmatis* mc^2^155 reference genome (NCBI accession: NC_008596) using BWA (v0.5.9) with default parameters [37]. Sequencing reads mapping to predicted open reading frames (ORFs) were quantified using HTSeq [38] based on the NCBI RefSeq annotation. Sequence counts for annotated ribosomal sequences and the tRNAs were filtered out prior to subsequent analysis. Differential expression analyses were performed in R (version 2.15.1) using the Bioconductor package, edgeR [39], and generalized linear modeling to account for gene expression dependencies on growth condition and on time. Differentially expressed genes were defined as genes with at least 2-fold change between samples, with *P*-value < 0.001 and false detection rate (FDR) value < 0.005. MDS plots were generated using the plotMDS function in R, selecting the 500 genes with the highest variation in expression (based on standard deviations calculated using all samples, including all growth conditions and time points) in the entire data set. COG enrichment analysis was performed using DAVID (v6.7) [40] with default parameters on the top 3000 significantly genes (based on P-value and FDR) among the data sets being compared.

## Additional files

Additional file 1: Table S1. List of *Mycobacterium smegmatis* mc^2^155 genes with statistically significant differential expression (log2 fold change > 1, *p* < 0.001) after starvation in PBS or PBS-Tween80 at 1, 3, 24 h and 14 d. (XLSX 2064 kb)
Additional file 2: Table S2. Functional classification of gene numbers up- and down-regulated during shock starvation in PBS-Tween80. (PDF 762 kb)
Additional file 3: Table S3. Functional classification of gene numbers up- and down-regulated during shock starvation in PBS. (PDF 1796 kb)
Additional file 4: Figure S1. Venn Diagrams showing overlap of significantly differentially expressed genes between SMRCs and LARCs at each time point. Upper diagrams indicate up-regulated genes and lower diagrams indicate down-regulated genes. The numbers of genes in each region of the diagrams are indicated. (PDF 29 kb)
Additional file 5: Figure S2. Venn Diagrams showing overlap of significantly differentially expressed genes among the four time points (1 h, 3 h, 24 h, 14 d) during SMRC and LARC development respectively. Upper diagrams indicate up-regulated genes and lower diagrams indicate down-regulated genes. The numbers of genes in each region of the diagrams are indicated. (PDF 322 kb)

## Abbreviations

COG: Clusters of orthologous groups
LARC: Large resting cell
MDS: Multidimensional scaling
OD: Optical density
PBS: Phosphate buffered saline
SMRC: Small resting cell
