# Supplementary material for: Developmental transcriptome of resting cell formation in Mycobacterium smegmatis
Source: BMC Genomics. 2016 Oct 26;17:837. doi: 10.1186/s12864-016-3190-4 (PMC5081680; doi:10.1186/s12864-016-3190-4)
Supplement: Additional file 2: Table S2. — Functional classification of gene numbers up- and down-regulated during shock starvation in PBS-Tween80. (PDF 762 kb) [file 12864_2016_3190_MOESM2_ESM.pdf]

Table S2 Functional classification of gene numbers up- and down-regulated during gentle starvation in PBS-Tween80

Functional classification [a]	Total	No. of genes [b] 1h Up	1h Down	3h Up	3h Down	24h Up	24h Down	14d Up	14d Down
Whole genome	6716	1185	1236	1212	1206	1509	1563	1476	1432
Energy production and conversion	510	65	76	71	88	98	77	108	84
Amino Acid metabolism and transport	500	107	71	117	82	106	85	97	72
Nucleotide metabolism and transport	103	26	27	21	16	16	29	14	22
Carbohydrate metabolism and transport	434	46	88	76	62	54	53	66	59
Coenzyme metabolism and transport	212	25	42	18	50	22	59	34	44
Lipid metabolism and transport	505	38	82	34	76	57	72	69	66
Inorganic ion transport and metabolism	282	34	55	35	53	48	49	54	65
Secondary metabolites biosynthesis, transport, catabolism	408	38	62	49	64	68	46	60	55
RNA processing and modification	18	4	0	5	2	4	2	2	2
Chromatin structure and dynamics	1	1	0	0	0	0	0	0	0
Translation, ribosomal structure and biogenesis	185	9	92	12	97	11	99	11	39
Transcription	600	113	51	97	48	79	42	95	51
Replication and repair	210	23	32	22	44	16	29	9	41
Cell cycle control, cell division, chromosome partitioning	30	6	6	7	2	1	5	0	4
Cell wall/membrane/envelop biogenesis	179	21	27	24	21	27	32	18	36
Protein export	10	0	1	1	1	2	1	1	1
Posttranslational modification, protein turnover, chaperones	139	23	21	16	23	16	30	18	29
Signal transduction mechanisms	187	37	15	55	10	54	11	50	19
Intracellular trafficking and secretion	28	3	7	2	7	1	9	1	5
Defense mechanisms	53	9	6	7	7	6	10	8	13
General functional prediction only	858	106	117	109	130	100	126	99	127
Function unknown	408	69	46	64	53	44	57	38	70
Not in COGs	856	300	181	269	185	290	176	263	198

a According to COG (clusters of orthologous groups) from NCBI NC_008596.
b Number of genes showing significant expression changes (>2-fold up- or down-regulation and a *P*-value<0.001 and false detection rate<0.005).
