# Supplementary material for: Developmental transcriptome of resting cell formation in Mycobacterium smegmatis
Source: BMC Genomics. 2016 Oct 26;17:837. doi: 10.1186/s12864-016-3190-4 (PMC5081680; doi:10.1186/s12864-016-3190-4)
Supplement: Additional file 3: Table S3. — Functional classification of gene numbers up- and down-regulated during shock starvation in PBS. (PDF 1796 kb) [file 12864_2016_3190_MOESM3_ESM.pdf]

**Table S3** Functional classification of gene numbers up- and down-regulated during shock starvation in PBS

| Functional classification [a] | Total | No. of genes [b] | | | | | | | |
|---|---|---|---|---|---|---|---|---|---|
| | | 1h | | 3h | | 24h | | 14d | |
| | | Up | Down | Up | Down | Up | Down | Up | Down |
| Whole genome | 6716 | 1000 | 1102 | 985 | 1091 | 1013 | 1015 | 1105 | 1091 |
| Energy production and conversion | 510 | 71 | 68 | 83 | 84 | 84 | 75 | 104 | 78 |
| Amino Acid metabolism and transport | 500 | 90 | 79 | 89 | 72 | 60 | 102 | 66 | 112 |
| Nucleotide metabolism and transport | 103 | 21 | 27 | 20 | 26 | 11 | 29 | 7 | 29 |
| Carbohydrate metabolism and transport | 434 | 60 | 57 | 64 | 66 | 61 | 54 | 56 | 57 |
| Coenzyme metabolism and transport | 212 | 19 | 50 | 22 | 47 | 22 | 42 | 24 | 38 |
| Lipid metabolism and transport | 505 | 48 | 78 | 36 | 78 | 79 | 60 | 106 | 59 |
| Inorganic ion transport and metabolism | 282 | 34 | 53 | 32 | 62 | 35 | 61 | 37 | 62 |
| Secondary metabolites biosynthesis, transport, catabolism | 408 | 52 | 57 | 43 | 58 | 64 | 41 | 83 | 45 |
| RNA processing and modification | 18 | 2 | 1 | 4 | 2 | 2 | 2 | 2 | 2 |
| Chromatin structure and dynamics | 1 | 0 | 0 | 0 | 0 | 0 | 0 | 0 | 0 |
| Translation, ribosomal structure and biogenesis | 185 | 12 | 97 | 9 | 95 | 7 | 90 | 8 | 67 |
| Transcription | 600 | 104 | 52 | 107 | 41 | 122 | 45 | 141 | 41 |
| Replication and repair | 210 | 20 | 55 | 30 | 38 | 11 | 38 | 8 | 34 |
| Cell cycle control, cell division, chromosome partitioning | 30 | 3 | 8 | 5 | 4 | 3 | 5 | 2 | 8 |
| Cell wall/membrane/envelop biogenesis | 179 | 29 | 26 | 22 | 26 | 17 | 28 | 13 | 32 |
| Protein export | 10 | 0 | 2 | 0 | 2 | 0 | 2 | 0 | 0 |
| Posttranslational modification, protein turnover, chaperones | 139 | 18 | 27 | 25 | 25 | 25 | 31 | 22 | 33 |
| Signal transduction mechanisms | 187 | 26 | 24 | 41 | 15 | 55 | 18 | 42 | 18 |
| Intracellular trafficking and secretion | 28 | 2 | 6 | 3 | 7 | 3 | 7 | 3 | 4 |
| Defense mechanisms | 53 | 11 | 9 | 7 | 7 | 8 | 9 | 7 | 10 |
| General functional prediction only | 858 | 121 | 106 | 109 | 113 | 103 | 110 | 115 | 115 |
| Function unknown | 408 | 59 | 52 | 63 | 52 | 58 | 64 | 44 | 61 |
| Not in COGs | 856 | 277 | 266 | 255 | 260 | 278 | 284 | 355 | 269 |

*a* According to COG (clusters of orthologous groups) from NCBI NC_008596.
*b* Number of genes showing significant expression changes (>2-fold up- or down-regulation and a *P*-value<0.001 and false detection rate<0.005).
