# Supplementary figures and images for: Developmental transcriptome of resting cell formation in Mycobacterium smegmatis
Source: BMC Genomics. 2016 Oct 26;17:837. doi: 10.1186/s12864-016-3190-4 (PMC5081680; doi:10.1186/s12864-016-3190-4)

1h

3h

24h

14d

## a) Up-regulated transcripts

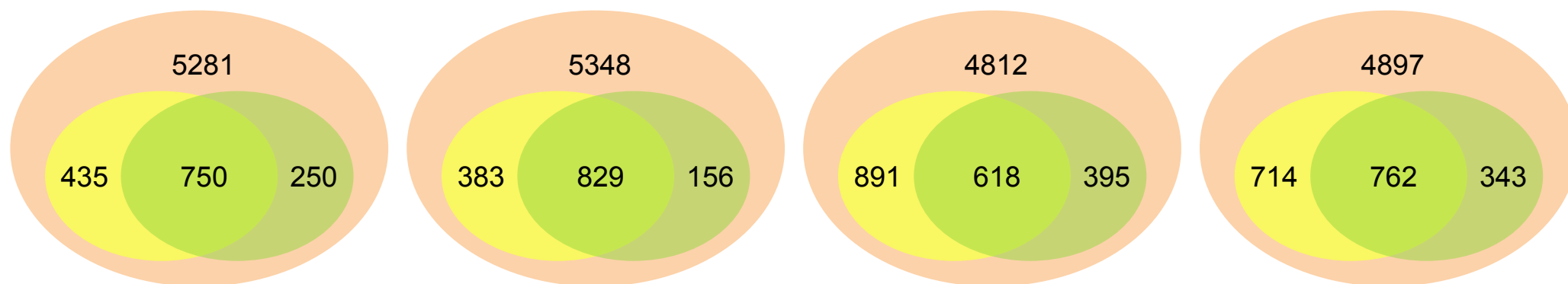

## b) Down-regulated transcripts

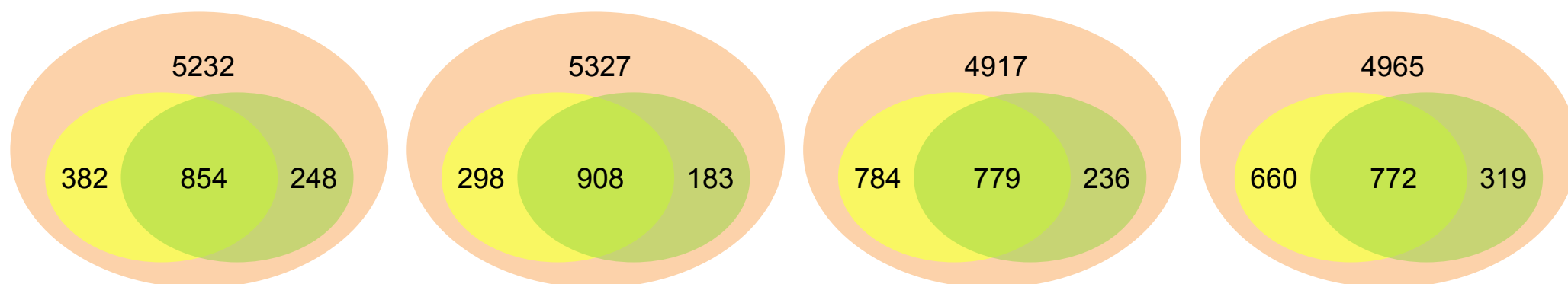

Total

SMRCs

LARCs

Supplement: Additional file 4: Figure S1. — Venn Diagrams showing overlap of significantly differentially expressed genes between SMRCs and LARCs at each time point. Upper diagrams indicate up-regulated genes and lower diagrams indicate down-regulated genes. The numbers of genes in each region of the diagrams are indicated. (PDF 29 kb) [file 12864_2016_3190_MOESM4_ESM.pdf]
