# Supplementary material for: Developmental transcriptome of resting cell formation in Mycobacterium smegmatis
Source: BMC Genomics. 2016 Oct 26;17:837. doi: 10.1186/s12864-016-3190-4 (PMC5081680; doi:10.1186/s12864-016-3190-4)
Supplement: Additional file 5: Figure S2. — Venn Diagrams showing overlap of significantly differentially expressed genes among the four time points (1 h, 3 h, 24 h, 14 d) during SMRC and LARC development respectively. Upper diagrams indicate up-regulated genes and lower diagrams indicate down-regulated genes. The numbers of genes in each region of the diagrams are indicated. (PDF 322 kb) [file 12864_2016_3190_MOESM5_ESM.pdf]

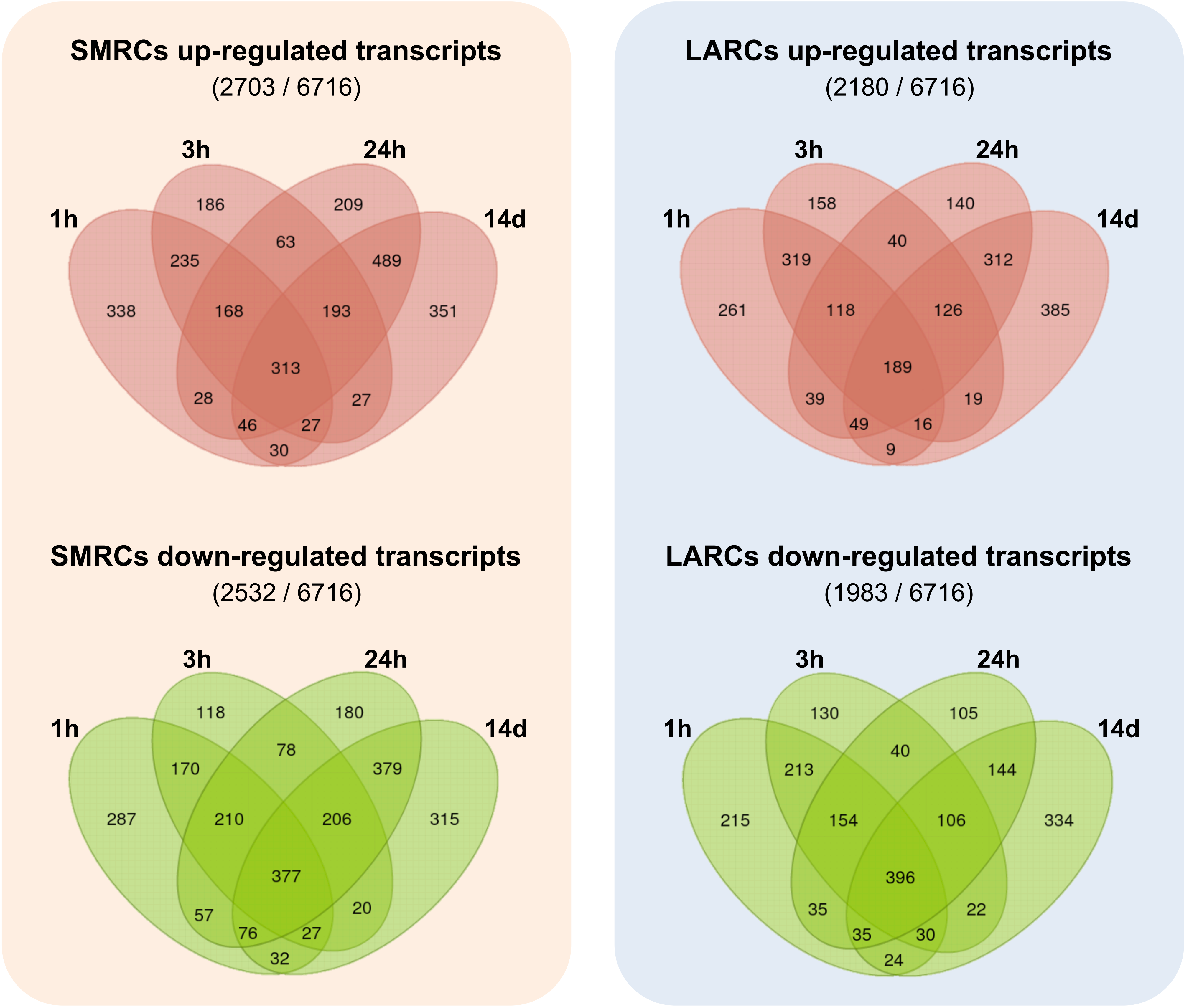

SMRCs up-regulated transcripts
(2703 / 6716)
3h
24h
1h
14d
186
209
63
235
489
338
168
193
351
313
28
27
46
27
30
LARCs up-regulated transcripts
(2180 / 6716)
3h
24h
1h
14d
158
140
40
319
312
261
118
126
385
189
39
19
49
16
9
SMRCs down-regulated transcripts
(2532 / 6716)
3h
24h
1h
14d
118
180
78
170
379
287
210
206
315
377
57
20
76
27
32
LARCs down-regulated transcripts
(1983 / 6716)
3h
24h
1h
14d
130
105
40
213
144
215
154
106
334
396
35
22
35
30
24
